# Inhibition of Soluble Epoxide Hydrolase Activity by Components of *Glycyrrhiza uralensis*

**DOI:** 10.3390/ijms24076485

**Published:** 2023-03-30

**Authors:** Jang Hoon Kim, Yun-Chan Huh, Mok Hur, Woo Tae Park, Youn-Ho Moon, Tae Il Kim, Yong Il Kim, Seon Mi Kim, Jeonghoon Lee, Ik Soo Lee

**Affiliations:** 1 Department of Herbal Crop Research, National Institute of Horticultural and Herbal Science, RDA, Eumseong 27709, Republic of Korea; jhkim53@korea.kr (J.H.K.);; 2 Km Covergence Research Division, Korea Institute of Oriental Medicien, Daejeon 34134, Republic of Korea

**Keywords:** *Glycyrrhiza uralensis*, soluble epoxide hydrolase, competitive inhibitor, noncompetitive inhibitor

## Abstract

Soluble epoxide hydrolase (sEH) is a target enzyme for the treatment of inflammation and cardiovascular disease. A *Glycyrrhiza uralensis* extract exhibited ~50% inhibition of sEH at 100 μg/mL, and column chromatography yielded compounds **1**–**11**. Inhibitors **1**, **4**–**6**, **9**, and **11** were non-competitive; inhibitors **3, 7**, **8**, and **10** were competitive. The IC_50_ value of inhibitor **10** was below 2 μM. Molecular simulation was used to identify the sEH binding site. Glycycoumarin (**10**) requires further evaluation in cells and animals.

## 1. Introduction

Epoxyeicosatrienoic acids (EETs) are derived from arachidonic acid, which is metabolized by cytochrome P450 (CYP) epoxygenase enzymes [1]. The CYP450 pathway yields four regioisomers of EETs: 5,6-EET, 8,9-EET, 11,12-EET, and 14,15-EET [2]. The EETs are autocrine- and paracrine-signaling lipids that exert antihypertensive, cardioprotective, renal-protective, vasodilative, pro-angiogenic, and metabolic/regulatory effects [3]. Growing evidence indicates that inflammatory conditions reduce the expression and activity of CYP450 enzymes [4], suggesting that EETs are potential, endogenous anti-inflammatory mediators. Experimentally, 11,12-EET prevented the tumor necrosis factor (TNF)-α-induced activation of nuclear factor (NF)-κB and increased vascular cell adhesion molecule-1 (VCAM-1) expression in endothelial cells [5,6]. EETs are rapidly metabolized by several means. In many tissues, the dominant activity is that of soluble epoxide hydrolase (sEH), which adds water to epoxides to generate the corresponding diols (dihydroxyeicosatrienoic acids, DHETs) [7]. Conversion of EETs to DHETs by sEH reduces the tissue levels of EETs and thus their anti-inflammatory effects. Tests of sEH inhibitors using various models of inflammation have supported the potent anti-inflammatory properties of such materials [8,9].

In particular, sEH is an enzyme mainly expressed in the liver, and alcohol-induced inflammation, injury, and steatosis were reduced in hepatic sEH-knockout mice [10]. Moreover, an sEH inhibitor, PTUPB, inhibited the expression of TNF-a, MCP-1, and IL-6 in non-alcoholic fatty liver disease mice [11]. Therefore, sEH inhibition is known as a target enzyme to reduce the inflammatory response in alcohol- or non-alcohol-induced inflammation in the liver [10,11].

Licorice (*Gancao*) prepared from the roots of *Glycyrrhiza* species (*Fabaceae*) is widely used to treat various human ailments [12]. The genus *Glycyrrhiza* contains approximately 30 species of perennial herbs, principally derived from Asia, Europe, North and South America, and Australia [13]. Three species, *Glycyrrhiza glabra* L., *G. inflata* Bat., and *G. uralensis* Fisch., are recognized by the Chinese Pharmacopoeia as sources of licorice [14]. During our continuing efforts to identify effective natural sEH inhibitors, we found that *G. uralensis* root extracts exhibited significant anti-sEH effects, and we isolated eleven active flavonoids. We here report the isolation of these materials, and their sEH inhibitory effects.

## 2. Results

### 2.1. Extract and Isolation of Materials

**Figure 1:**
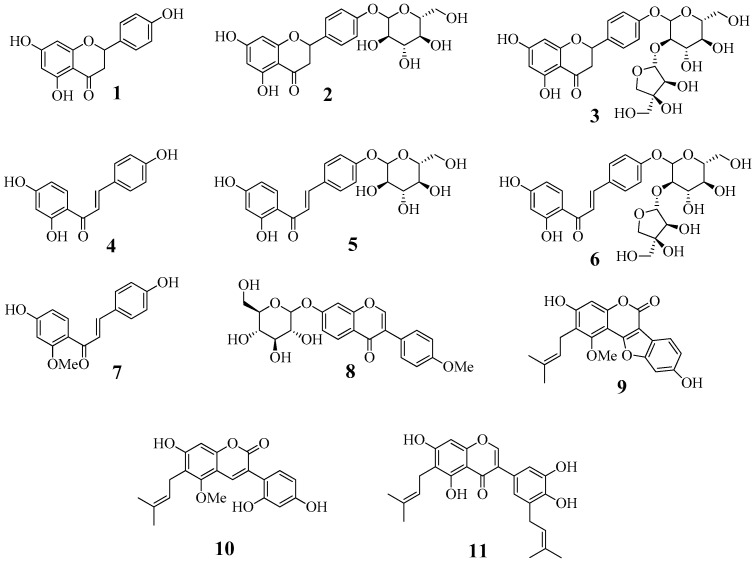
The structure of eleven compounds, **1**−**11,** from the roots of *G. uralensis*. (**1**: liquiritigenin, **2**: liquiritin, **3**: liquiritin apioside, **4**: isoliquiritigenin, **5**: isoliquiritin, **6**: isoliquiritin apioside, **7**: 2′-methoxyisoliquiritigenin, **8**: ononin, **9**: glycyrol, **10**: glycycoumarin, **11**: isoangustone A).

An ethanol extract of *G. uralensis* roots that significantly inhibited sEH activity (50.6 ± 0.8% inhibition at 100 µg/mL) was suspended in water and successively partitioned using CHCl_3_ and EtOAc. These fractions were subjected to a series of chromatographic steps to isolate flavonones **1**–**3** [15], chalcones **4**–**7** [16], and an isoflavonoid **8** [15] from the EtOAc-soluble fraction, and coumestan **9** [16], prenyl-coumarine **10** [16], and prenyl-isoflavonoid **11** [16] from the CHCl_3_-soluble fraction. The chemical structures of these isolates were identified by comparing their physicochemical and spectral data to those of the literature (Figure 1, Figures S1–S11)

### 2.2. Inhibition of sEH by the Isolated Compounds

**Figure 2:**
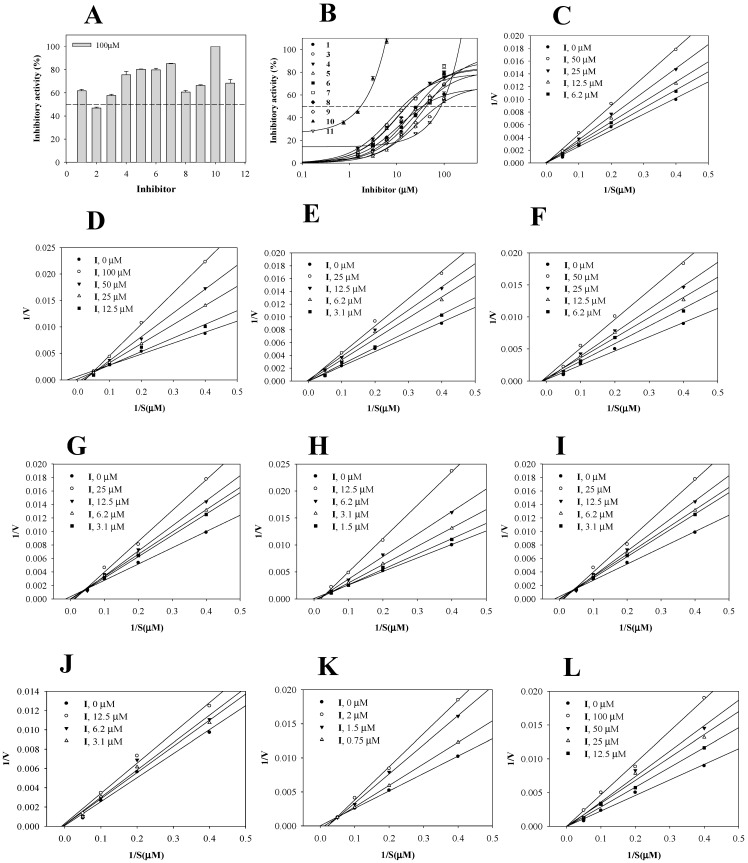
The inhibitory activity of compounds **1**−**11** at 100 μM (**A**) and at a variety of concentrations (**B**) in terms of sEH. Lineweaver–Burk plots of the inhibitors **1** (**C**) and **3**−**11** (**D**–**L**) in terms of sEH.

The inhibitory effects of the isolated compounds **1**–**11** against sEH in vitro were determined using a modification of an earlier method, employing commercially available AUDA, a known inhibitor of sEH, as the reference standard. Although the sEH inhibitory activities of the isolates were less potent than those of AUDA (IC_50_, 22 ± 0.8 nM), all tested compounds except **2** exhibited a considerable inhibition (Equation (1)) of sEH activity, with IC_50_ values (Equation (2)) from 1.9 ± 0.2 to 85.7 ± 1.2 µM (Figure 2A,B, Table 1).

**Figure 3:**
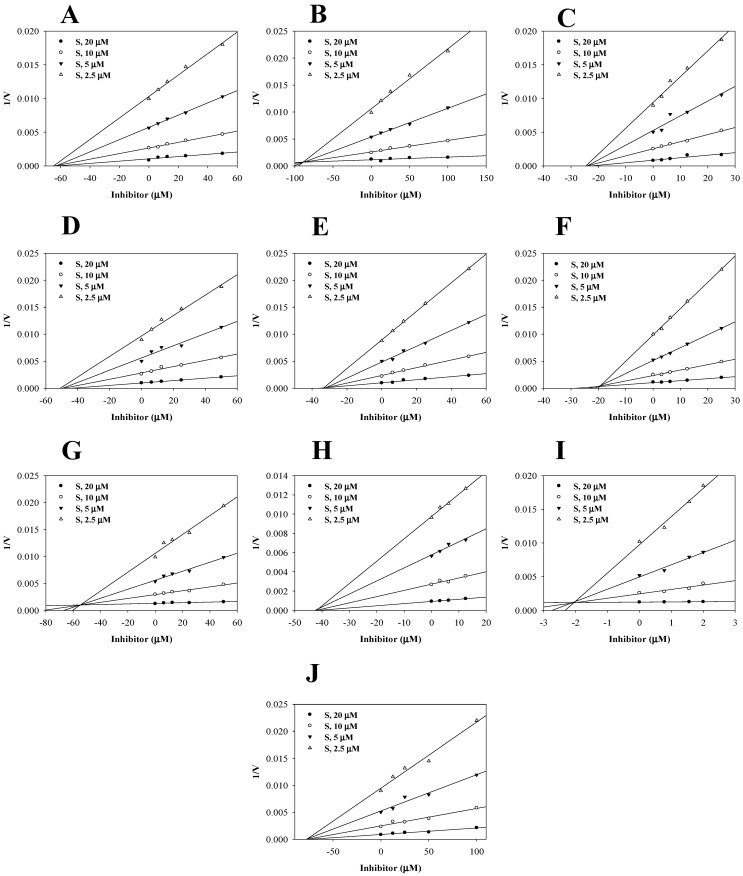
Dixon plots of the inhibitors **1** (**A**) and **3**–**11** (**B**–**J**) in terms of sEH.

To further characterize the inhibitory behaviors, enzyme kinetics were studied in the presence of various concentrations of 10 active compounds (**1** and **3**–**11**). As shown by the Lineweaver–Burk plot (Figure 2C–L), the x-intercept (−1/Km) was unaffected by increasing concentrations of **1**, **4**–**6**, **9**, and **11**, but 1/Vmax gradually increased, indicating that these compounds were noncompetitive inhibitors. On the other hand, compounds **3**, **7**, **8**, and **10** were competitive inhibitors, with different Km values. Dixon plot analysis indicated that the inhibition constants (*k*_i_ values) of compounds **1** and **3**–**11** ranged from 2.0 to 89.1 µM (Figure 3A–J, Table 1).

### 2.3. Molecular Docking

**Figure 4:**
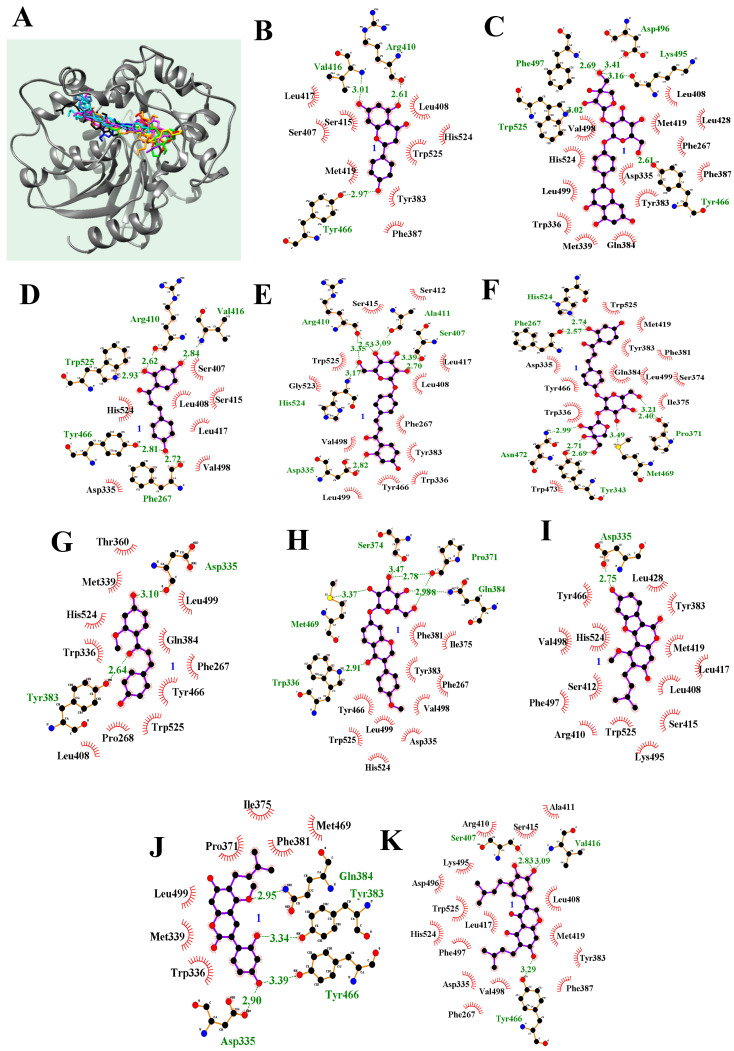
Predicted docking poses of inhibitors **1** and **3**−**11** (**A**) with sEH. The green dot line hydrogen bonds of inhibitors with amino acids of sEH (**B**–**K**).

To predict the binding of the sEH inhibitors **1** and **3**–**11** to sEH, we performed molecular docking analyses guided by the enzyme kinetic data. The noncompetitive inhibitors **1**, **4**–**6**, **9**, and **11** were subjected to blind docking, and the competitive inhibitors **3**, **7**, **8**, and **10** were subjected to docking at the active site. As indicated in Figure 4 and Table 2, compounds **1** and **3**–**11** bound stably to she, with autodock scores of −8.25, −9.38, −8.51, −10.06, −9.15, −7.94, −9.22, −9.40, −8.57, and −10.38 kcal/mol, respectively. All the compounds formed hydrogen bonds with amino acid residues of she, both in and around the binding pocket. The results are described in detail in Figure 4 and Table 2.

### 2.4. Molecular Dynamics 

**Figure 5:**
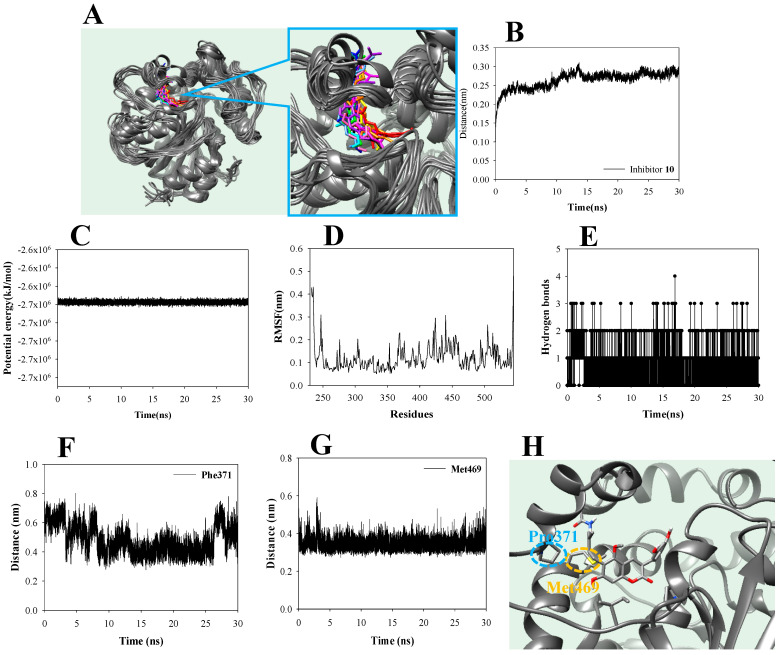
The superposition of inhibitors **10** with sEH across30 ns (red: 0 ns, orange: 3 ns, yellow: 6 ns, green: 9 ns, cyan: 12 ns, blue: 15 ns, cornflower blue: 18 ns, purple: 21 ns, hot pink: 24 ns, magenta: 27 ns, black: 30 ns) (**A**). The RMSD (**B**), potential energy (**C**), RMSF (**D**), and hydrogen bonds (**E**) of the simulation. The distance of key residues (**H**) of inhibitor **10** from sEH (**F**,**G**).

A dynamic study was performed, based on the docking results, to predict the binding of **10** to sEH. As shown in Figure 5A, the complex of compound **10** and sEH exhibited stable fluidic motion. The inhibitor initially bound to the active site, but towards the right pocket (Pro371–Met469), commencing at 3 ns. Compound **10** evidenced root mean square deviation (RMSD) values of about 3 Å, and a potential energy of approximately −2.7 × 106 kJ/mol across the duration of the simulation (Figure 5B,C). The enzyme residues affected by inhibitor 10 showed fluidities below 3 Å of the root mean square fluctuations (RMSFs) (Figure 5D), which were maintained principally by one, but occasionally two to four, hydrogen bonds (Figure 5E). Inhibitor **10** maintained a distance within 4 Å from the active site, and could thus bind Phe371 and Met469 (Figure 5F–H).

## 3. Discussion

sEH inhibition elevates EET levels, which would be expected to elicit a variety of beneficial biological effects [17] that effectively treat atherosclerosis, diabetes, hypertension, lung disease, pain, inflammation, immune disorders, and other diseases [18]. Thus, sEH is a potential pharmaceutical target. Secondary metabolites of medicinal plants play important roles in drug discovery by providing lead scaffolds that can then be optimized by synthetic and medicinal chemists. So far, research has been conducted to develop sEH inhibitors from natural products, and, as a result, flavonoids [19], triterpenoids [20], and macamides [21] are representative compounds.

During our continuous efforts to identify potent sEH inhibitors in medicinal plants, we focused on the polyphenols of *G. uralensis* roots; these evidence diverse biological activities. *G. uralensis* is one of the most popular Chinese medicinal herbs, and has been shown to exhibit various pharmacological activities, including antidiabetic, anti-inflammatory, antioxidant, antiviral, cytotoxic, skin-whitening, hepatoprotective, and cholinergic properties [22,23,24]. The roots of this plant contain various secondary metabolites, and include different classes of phenolic compounds, such as flavonoids, chalcones, coumarins, and triterpenoid saponins [25]. Of these, flavonoids have frequently been reported to show major anti-inflammatory, antibacterial, antimicrobial, antioxidant, and cytotoxic activities [25]. We thus screened a licorice ethanol extract in terms of sEH inhibition, which showed 50.6 ± 0.8% inhibition at 100 μg/mL. However, the value for glycyrrhizin, the main compound of *G. uralensis*, was only 18.4 ± 0.5% at 100 μM; we isolated eleven polyphenol components **1**–**11** from *G. uralensis* in a search for potential inhibitors. Except for coumarin **10**, chalcone compounds **4**–**7** exhibited higher efficacies than flavonoids **1**–**3**, isoflavonoids **8** and **11**, and coumestan **9**. In particular, compounds **1**, **4**, and **7,** in the form of aglycones, showed better efficacies than glycosides **2**, **3**, **5**, and **6**. In addition, glycosides with two sugars (**3** and **6**) were more effective than those with one sugar (**2** and **5**). When comparing the biological activities of **4**–**7** to **1**–**3**, as well as the activity of **10** to **9**, inhibitors **4**–**7** and **10,** with flexible carbon–carbon bonds, showed better inhibitory activities. In particular, the potential inhibitor **10** remained ~3.5 Å distant from Pro371 and Met469 over the simulation time. This inhibitor remained bound in a mobile fashion to the active site and the adjacent pocket. Flexible compounds would be expected to more easily bind to sEH than inflexible materials. 

## 4. Materials and Methods

### 4.1. General Experimental Procedures

Chromatographic separations employed thin-layer chromatography (TLC) on glass plates pre-coated with silica gel 60 F254 and silica gel RP-18 F254 (20 cm × 20 cm; Merck, Darmstadt, Germany); bands were visualized under ultraviolet light of 254 nm. The color reagent was 10% (*v*/*v*) ethanol–sulfuric acid. Column chromatography employed 230–400-mesh silica gel 60 (Merck, Darmstadt, Germany), 12-nm ODS-A (YMC, Kyoto, Japan), and Sephadex LH-20 (GE HealthCare, Chicago, IL, USA) columns. NMR experiments were performed using Bruker Avance III 400 MHz spectrometers (Bruker, Billerica, MA, USA). Mass spectral data were obtained using an LCMS-2020-EV platform, featuring electrospray ionization (Shimadzu, Kyoto, Japan). Glycyrrhizin (PHL89217), Tris (catalog no. B9754) and bovine serum albumin (BSA) (A8806) were purchased from Sigma-Aldrich (St. Louis, MO, USA). Human recombinant sEH (10011669), PHOME (10009134), and AUDA (10007972) were purchased from Cayman Chemical (Ann Arbor, MI, USA).

### 4.2. Extract Treatment

Dried *G. uralensis* roots (5 kg) were extracted with EtOH three times (10 L each time) at room temperature for 7 days, after maceration, filtration, and concentration, to yield an EtOH extract (240 g) that was suspended in H_2_O (4 L), and this was partitioned successively with CHCl_3_ (3 × 4.0 L) and EtOAc (3 × 4.0 L) to yield CHCl_3_- (80 g), EtOAc- (87 g), and water-soluble fractions, respectively. The EtOAc-soluble fraction (87 g) was subjected to silica gel column chromatography (70–230 μm, 60 × 9.5 cm), featuring elution, with a gradient of CHCl_3_–MeOH (9:1→1:9) that generated seven fractions (A–G). Fraction C was chromatographed on a silica gel column (230–400 mesh, 60 × 6.5 cm) using a CHCl_3_–MeOH gradient solvent (8:2→1:9) that yielded five subfractions (C1–C5). Fraction C2 was further chromatographed on an ODS column (50 × 4.5 cm) eluted with a MeOH–H_2_O gradient system (70:30→90:10 *v*/*v*) that yielded compounds **1** (120 mg), **2** (705 mg), and **3** (117 mg). Fraction C4 was purified on a Sephadex LH column (100 × 3.0 cm) eluted with an MeOH isocratic solvent system, in order to yield compounds **4** (50 mg) and **5** (40 mg). Chromatography of fraction D on a silica gel column (230–400 mesh, 60 × 6.5 cm), using a CHCl_3_–MeOH gradient solvent system (8:2→1:9), yielded three subfractions (D1–D3). Fraction D2 was further purified on an ODS column (50 × 3.5 cm) eluted with a MeOH–H_2_O gradient solvent system (40:60→80:20), in order to yield compounds **6** (55 mg) and **7** (12 mg). Fraction D3 was applied to the same ODS column and eluted with a MeOH–H_2_O gradient solvent system (40:60→70:30), in order to yield compound **8** (50 mg). The CHCl_3_-soluble fraction (80 g) was subjected to silica gel column chromatography (70–230 μm, 60 × 9.5 cm), eluted with a gradient solvent system of CHCl_3_–MeOH (10:0→0:10), and yielded four fractions (A–D). Fraction B was then chromatographed on a silica gel column (230–400 mesh, 60 × 6.5 cm), eluted with a CHCl_3_–MeOH gradient solvent system (9:1→1:9), and yielded three subfractions (B1–B3). Fraction B2 was further purified on an ODS column (50 × 3.0 cm) eluted with a MeOH–H_2_O gradient solvent system (40:60→80:20) that yielded compounds **9** (6 mg), **10** (53 mg), and **11** (21 mg).

### 4.3. sEH Assay

To evaluate inhibitory activities, 130 μL of an sEH solution in 25 mM bis-Tris–HCl buffer (pH 7.0), with 0.1% BSA, was added to 20 μL of a putative inhibitor dissolved in MeOH. Next, 50 μL of a substrate was added, and the mixture was incubated at 37 °C to allow for sEH hydrolysis. Product development was monitored (330 nm excitation wavelength and 465 nm emission wavelength) for approximately 40 min. The inhibitory activity was calculated as follows:

Inhibitory activity (%) = [(ΔC − ΔI)/ΔC] × 100(1)

where ΔC and ΔI are the optical densities of the control and inhibitor tubes, respectively, after 40 min, and:

y = y0 + (a × x/b + x)(2)

where y0 is the minimum y-axis value, a is the difference between the maximum and minimum values, and b is the x value at 50% of the a value.

### 4.4. Molecular Docking

To dock ligands to receptors, two three-dimensional (3D) ligand structures were prepared and minimized using Chem3D Pro (CambridgeSoft, Cambridge, MA, USA). The receptor protein structure coded in 3ANS was downloaded from the RCSB data bank. Only the A-chain of the enzyme is involved in docking; we did not evaluate the B-chain. Water and 4-cyano-N-[(1S,2R)-2-phenylcyclopropyl]-benzamide were excluded from the A-chain. The revised A-chain was hydrogenated using AutoDockTools (Scripps Research, La Jolla, CA, USA); the Gasteiger charge model was then applied. Flexible ligand docking was evaluated using a torsion tree that detected torsion roots and rotatable bonds. The grid box sizes were 126 × 126 × 126 at 0.375 Å and 60 × 60 × 60 (center grid box: x, 24.612; y, 26.057; z, 117.11) for blinded docking and ligand docking to the active site, respectively. Molecular docking was evaluated using a Lamarckian algorithm and the maximum possible evaluation number. The resulting values were calculated and visualized using AutoDockTools, Chimera ver. 1.14 (San Francisco, CA, USA), and LIGPLOT (European Bioinformatics Institute, Hinxton, UK).

### 4.5. Molecular Dynamics

3D ligand structures were built using the GlycoBioChem server. The sEH Gro was developed using the GROMOS96 45a3 force field. All complexes were surrounded by water molecules (cubic size 12 × 12 × 12) and six Cl anions. The energy level attained 10.0 kJ/mol via steepest descent minimization. Each inhibitor–sEH complex was sequentially subjected to NVT equilibration at 300 K, NPT and Particle Mesh Ewald evaluation of long-range electrostatics at 1 bar, and MD simulation for 30 ns.

### 4.6. Statistical Analysis

All measurements were performed in triplicate (three independent experiments) and the results are means ± standard errors of the means (SEMs). The results were compared using Sigma Plot 10.0 (Systat Software Inc., San Jose, CA, USA).

## 5. Conclusions

Bioactivity screening revealed that an ethanol extract of *G. uralensis* roots inhibited sEH, but the main compound, glycyrrhizin, lacked such an effect. In this study, eleven polyphenol compounds, **1**–**11,** were isolated from *G. uralensis*. Among them, compounds **1**, **3**–**9**, and **11** were found to exert moderate inhibitory effects, and inhibitor **10** showed a potent effect, with an IC_50_ value of 1.9 ± 0.2 μM. Molecular simulation was used to study the binding of **1**, and **3**–**11** to sEH and it was found that the potential inhibitor **10** maintained a stable fluidic bond with the enzyme. Therefore, of the polyphenols, compound **10** was the strongest sEH inhibitor.

## Figures and Tables

**Table 1:** The inhibitory activity and enzyme kinetics of inhibitors from the roots of *G. uralensis*.

	Inhibitory Activity at 100 μM (%) ^a^	IC_50_ (μM)	Binding Mode (*k*_i_, μM)
**1**	61.5 ± 1.2	40.1 ± 1.2	Noncompetitive (63.1)
**2**	46.8 ± 0.8	N.T. ^b^	-
**3**	57.6 ± 1.1	72.3 ± 0.5	Competitive (89.1)
**4**	75.6 ± 2.9	17.6 ± 0.6	Noncompetitive (24.2)
**5**	80.2 ± 0.3	25.8 ± 0.2	Noncompetitive (48.8)
**6**	79.3 ± 1.4	22.2 ± 0.1	Noncompetitive (32.7)
**7**	85.1 ± 0.3	16.2 ± 0.3	Competitive (19.8)
**8**	60.7 ± 1.3	35.7 ± 0.7	Competitive (54.4)
**9**	69.5 ± 0.5	42.5 ± 1.5	Noncompetitive (41.1)
**10**	>100	1.9 ± 0.2	Competitive (2.0)
**11**	68.4 ± 3.1	85.7 ± 1.2	Noncompetitive (75.6)

EtOH extract: 50.6 ± 0.8% inhibitory activity at 100 μg/mL; Glycyrrhizin showed 18.4 ± 0.5% inhibitory activity at 100 μM; AUDA was used as a positive control (IC50: 22 ± 0.8 nM); ^a^ Compounds were tested three times. ^b^ Not tested.

**Table 2:** Molecular docking of sEH with inhibitors derived from the roots of *G. uralensis*.

	Autodock Score (kcal/mol)	Hydrogen Bonds
**1**	−8.25	Arg410 (2.61), Val416 (3.01), Tyr466 (2.97)
**3**	−9.38	Lys495 (3.16), Phe497 (2.69), Trp525 (3.02). Tyr466 (2.61)
**4**	−8.51	Val416 (2.84), Arg410 (2.62), Trp525 (2.93) Tyr466 (2.81), Phe267 (2.72)
**5**	−10.06	Asp335 (2.82), Ser407 (2.70), Ala411 (3.09), His524 (3.17) Arg410 (2.35, 3.35)
**6**	−9.15	His524 (2.74), Phe267 (2.57), Asn472 (2.99) Tyr343 (2.71, 2.69), Pro371 (2.40, 3.23)
**7**	−7.94	Asp335 (3.10), Tyr383 (2.64)
**8**	−9.22	Pro371 (2.78, 2.98), Gln384 (2.98), Trp336 (2.91)
**9**	−9.4	Asp335 (2.75)
**10**	−8.57	Gln384 (2.95), Tyr383 (3.34), Asp335 (2.90)
**11**	−10.38	Asp335 (2.50)

## Data Availability

Data are contained within the article and Supplementary Material.

## Supplementary Materials

The following supporting information can be downloaded at: https://www.mdpi.com/article/10.3390/ijms24076485/s1.
