# Supplementary material for: Inhibition of Soluble Epoxide Hydrolase Activity by Components of Glycyrrhiza uralensis
Source: Int J Mol Sci. 2023 Mar 30;24(7):6485. doi: 10.3390/ijms24076485 (PMC10095357; doi:10.3390/ijms24076485)
Supplement: Supplementary file 1 [file ijms-24-06485-s001.zip › ijms-2281726-supplementary.pdf]

Inhibition of soluble epoxide hydrolase activity by components of *Glycyrrhiza uralensis*

Jang Hoon Kim [1], Yun-Chan Huh [1], Mok Hur [1], Woo Tae Park [1], Youn-Ho Moon [1], Tae Il Kim [1], Yong Il Kim [1], Seon Mi Kim [1], Jeonghoon Lee [1,*] and Ik Soo Lee [2,*]

[1] Department of Herbal Crop Research, National Institute of Horticultural and Herbal Science, RDA, Eumseong 27709, Republic of Korea

[2] Km Covergence Research Division, Korea Institute of Oriental Medicien, Daejeon 34134, Republic of Korea

* Correspondence: artemisia@korea.kr (J.L.); knifer48@kiom.re.kr (I.S.L.); Tel.: +82-42-871-5670 (J.L.); +82-42-868-9462 (I.S.L.)

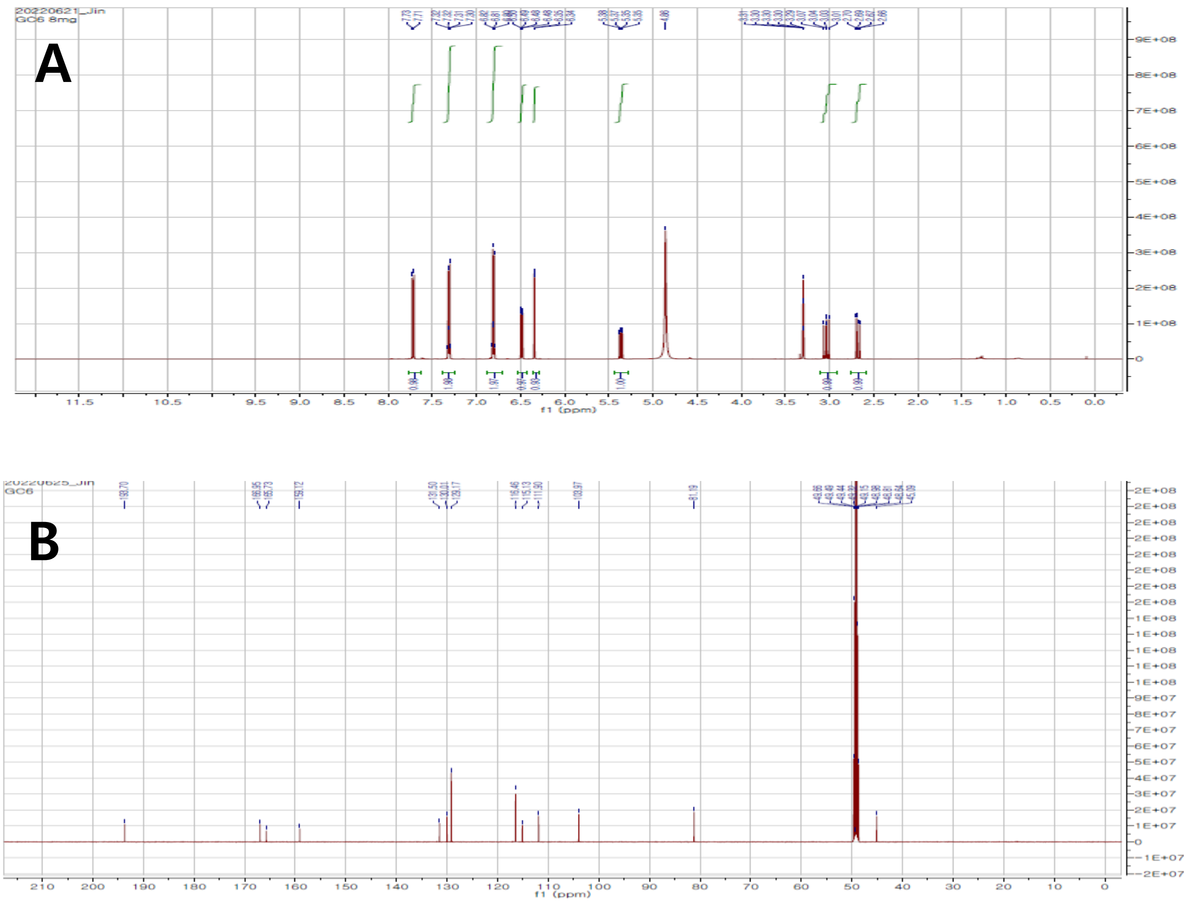

Figure S1. ^{1}H NMR(A) and ^{13}C NMR(B) spectra of compound **1** in MeOD-*d*$_4$ (400 MHz for ^{1}H NMR, 100 MHz for ^{13}C NMR)

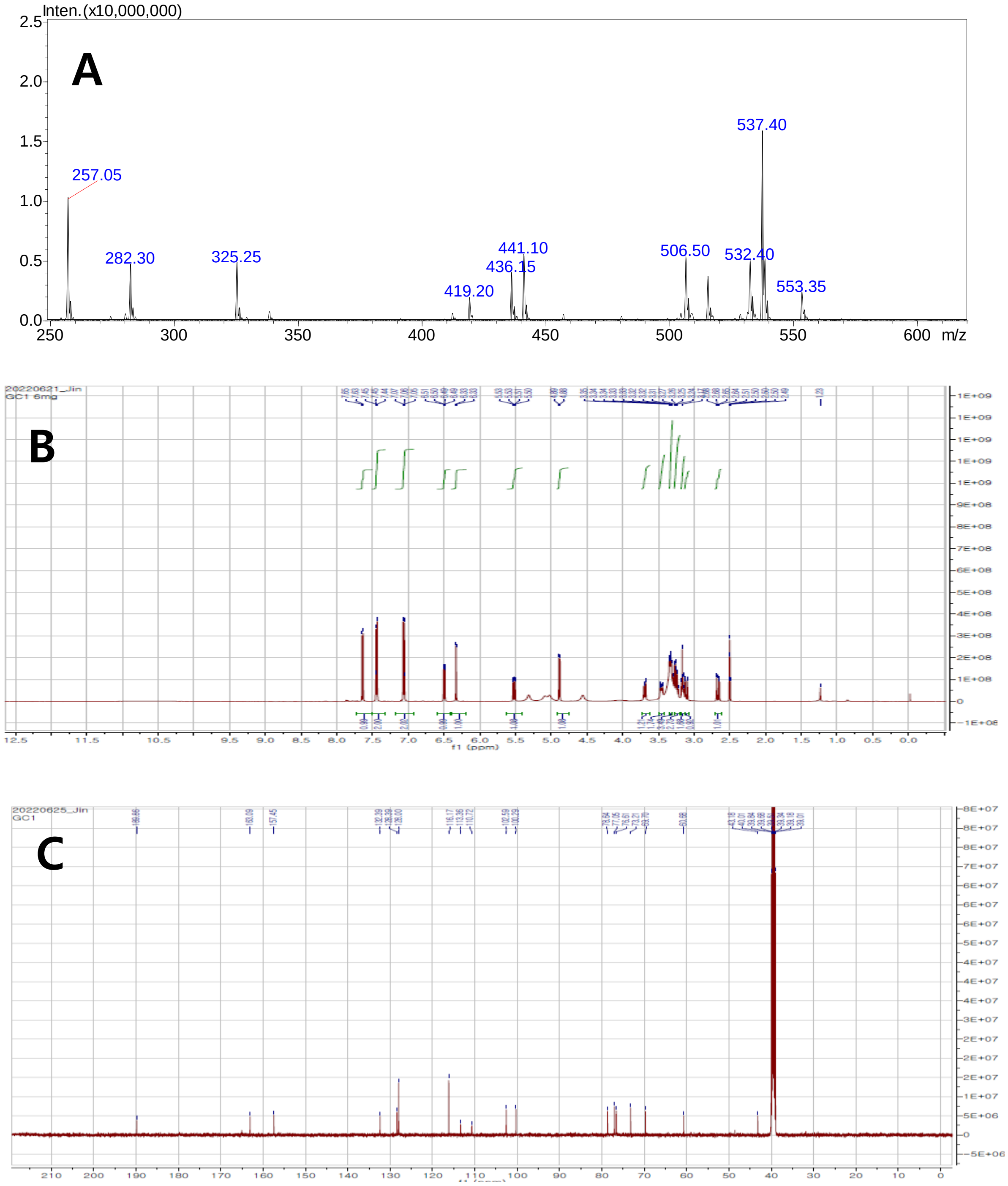

Figure S2. LC-MS(A), ^{1}H NMR(B) and ^{13}C NMR(C) spectra of compound **2** in MeOD-d_4 (400 MHz for ^{1}H NMR, 100 MHz for ^{13}C NMR)

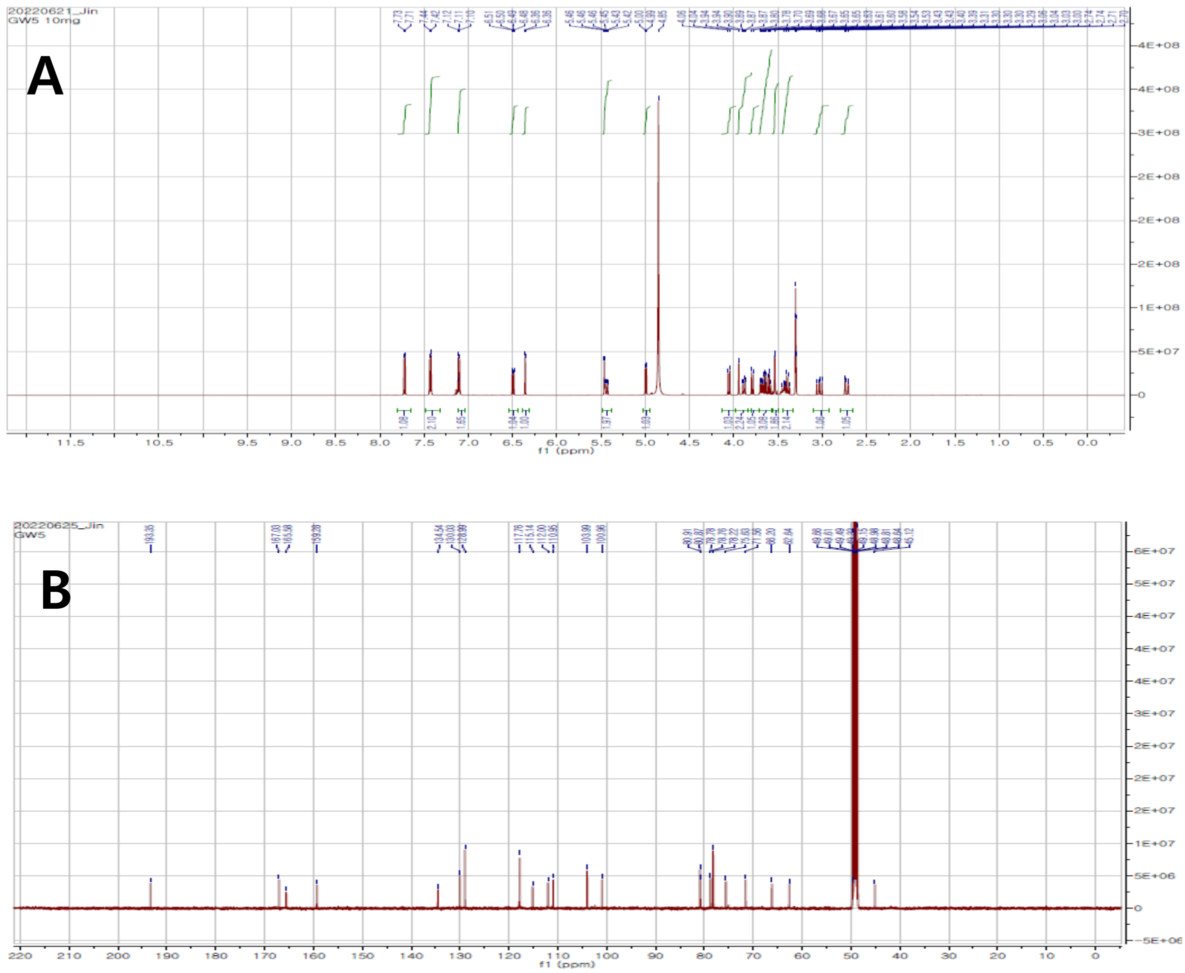

Figure S3. ^{1}H NMR(A) and ^{13}C NMR(B) spectra of compound **3** in MeOD-*d*4 (400 MHz for ^{1}H NMR, 100 MHz for ^{13}C NMR)

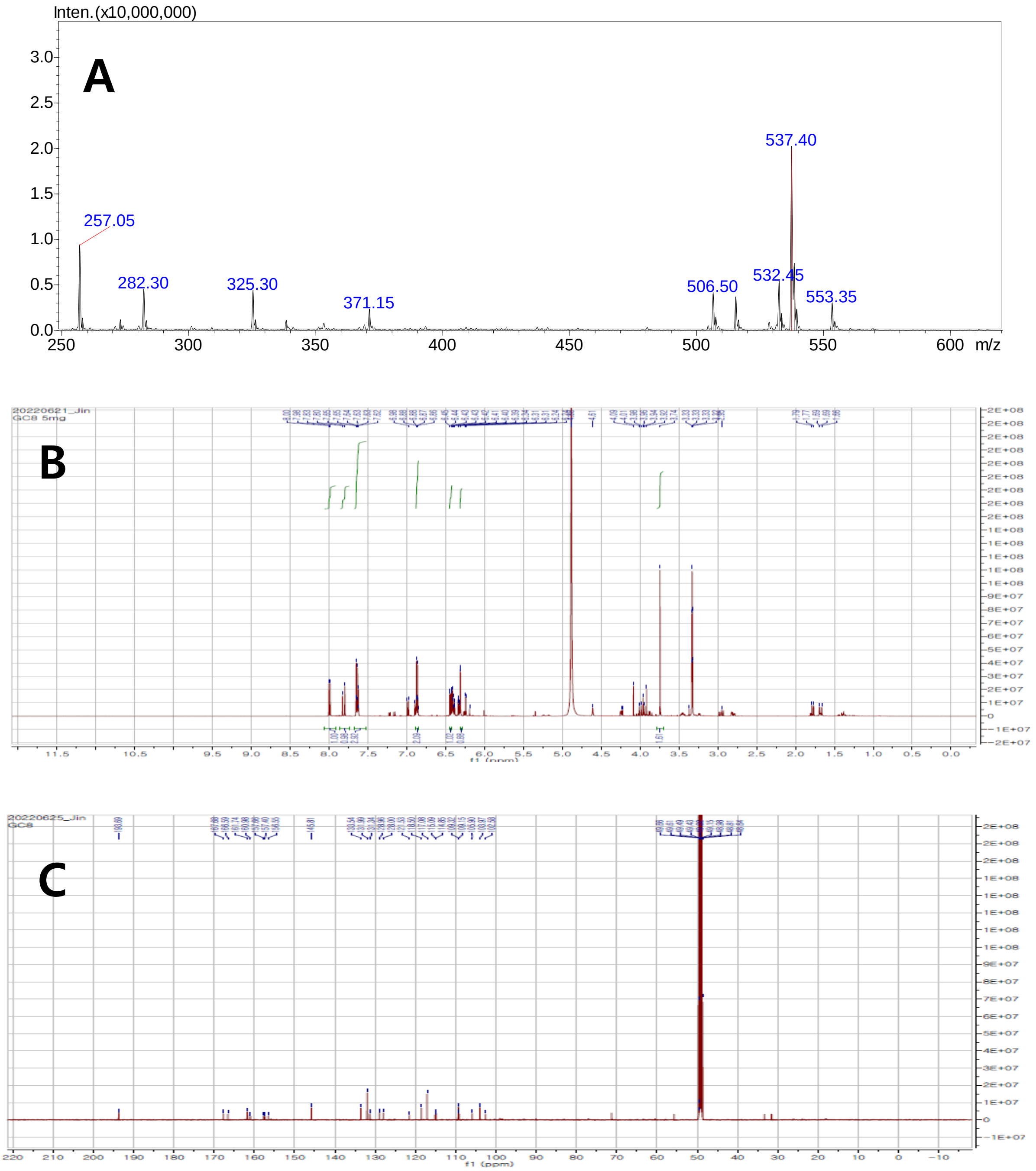

Figure S4. LC-MS(A), ^{1}H NMR(B) and ^{13}C NMR(C) spectra of compound **4** in MeOD-d_4 (400 MHz for ^{1}H NMR, 100 MHz for ^{13}C NMR)

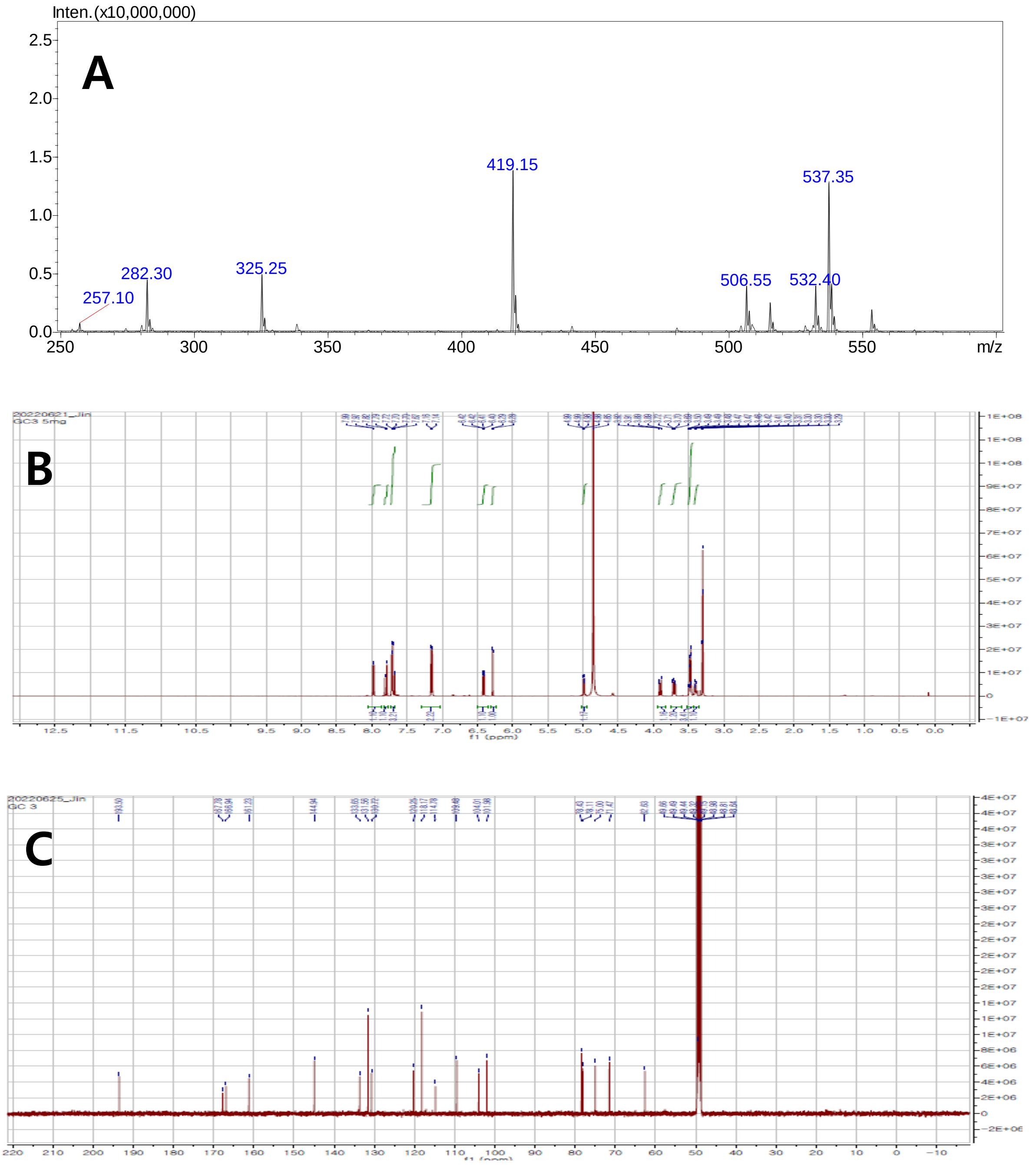

Figure S5. LC-MS(A), ^{1}H NMR(B) and ^{13}C NMR(C) spectra of compound **5** in MeOD-d_4 (400 MHz for ^{1}H NMR, 100 MHz for ^{13}C NMR)

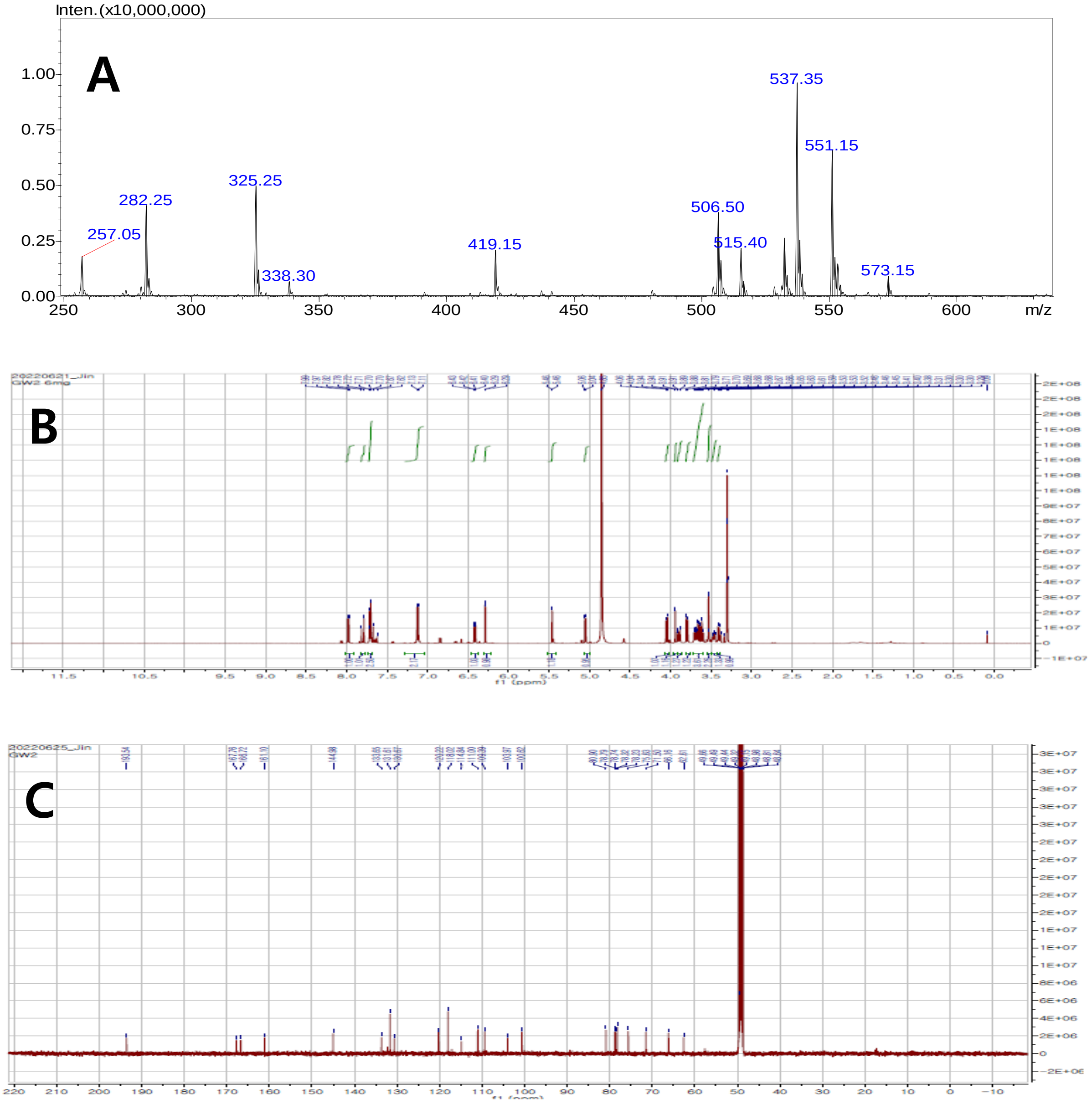

Figure S6. LC-MS(A), ^{1}H NMR(B) and ^{13}C NMR(C) spectra of compound **6** in MeOD-d_4 (400 MHz for ^{1}H NMR, 100 MHz for ^{13}C NMR)

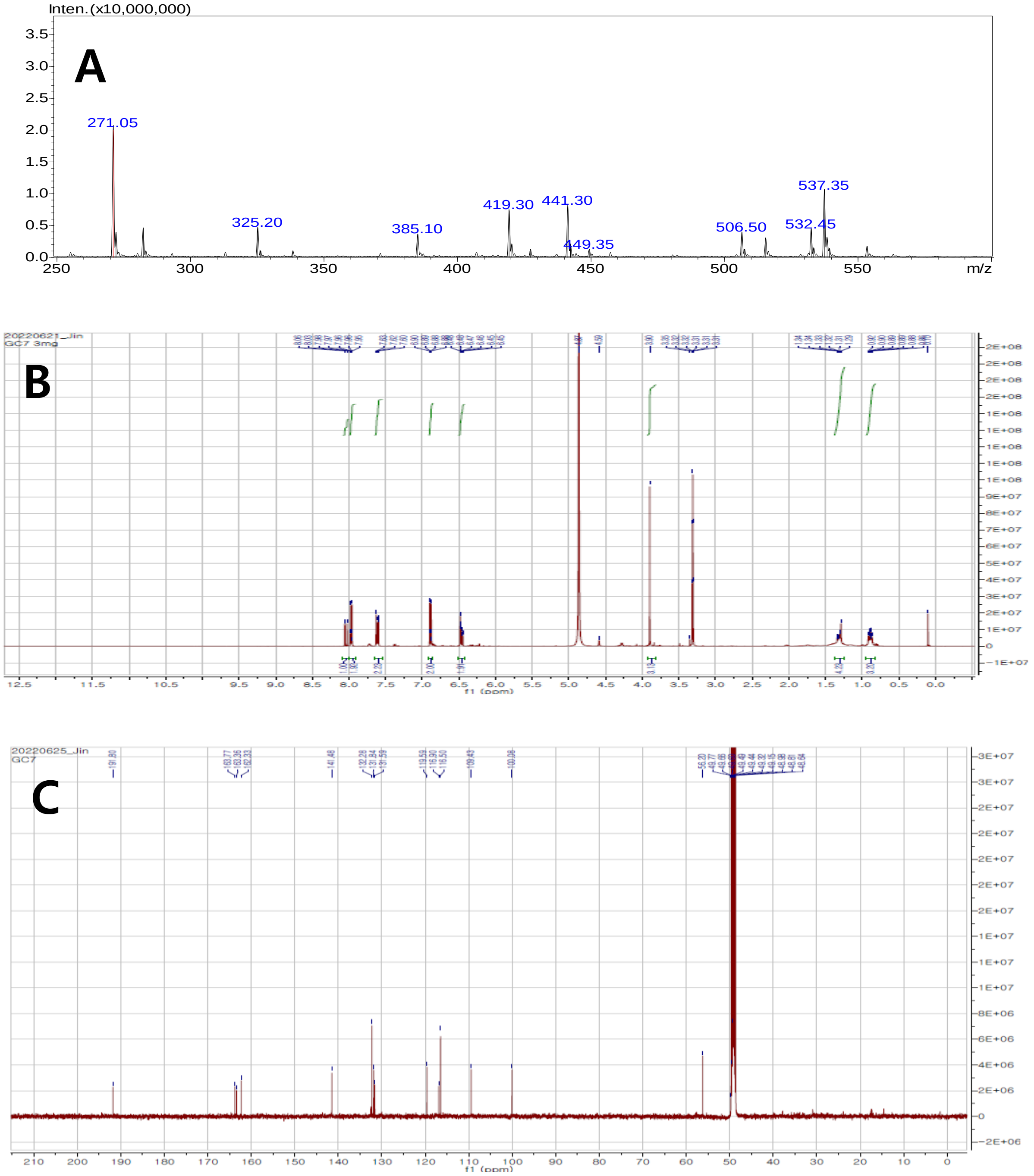

Figure S7. LC-MS(A), ^{1}H NMR(B) and ^{13}C NMR(C) spectra of compound **7** in MeOD-*d*$_4$ (400 MHz for ^{1}H NMR, 100 MHz for ^{13}C NMR)

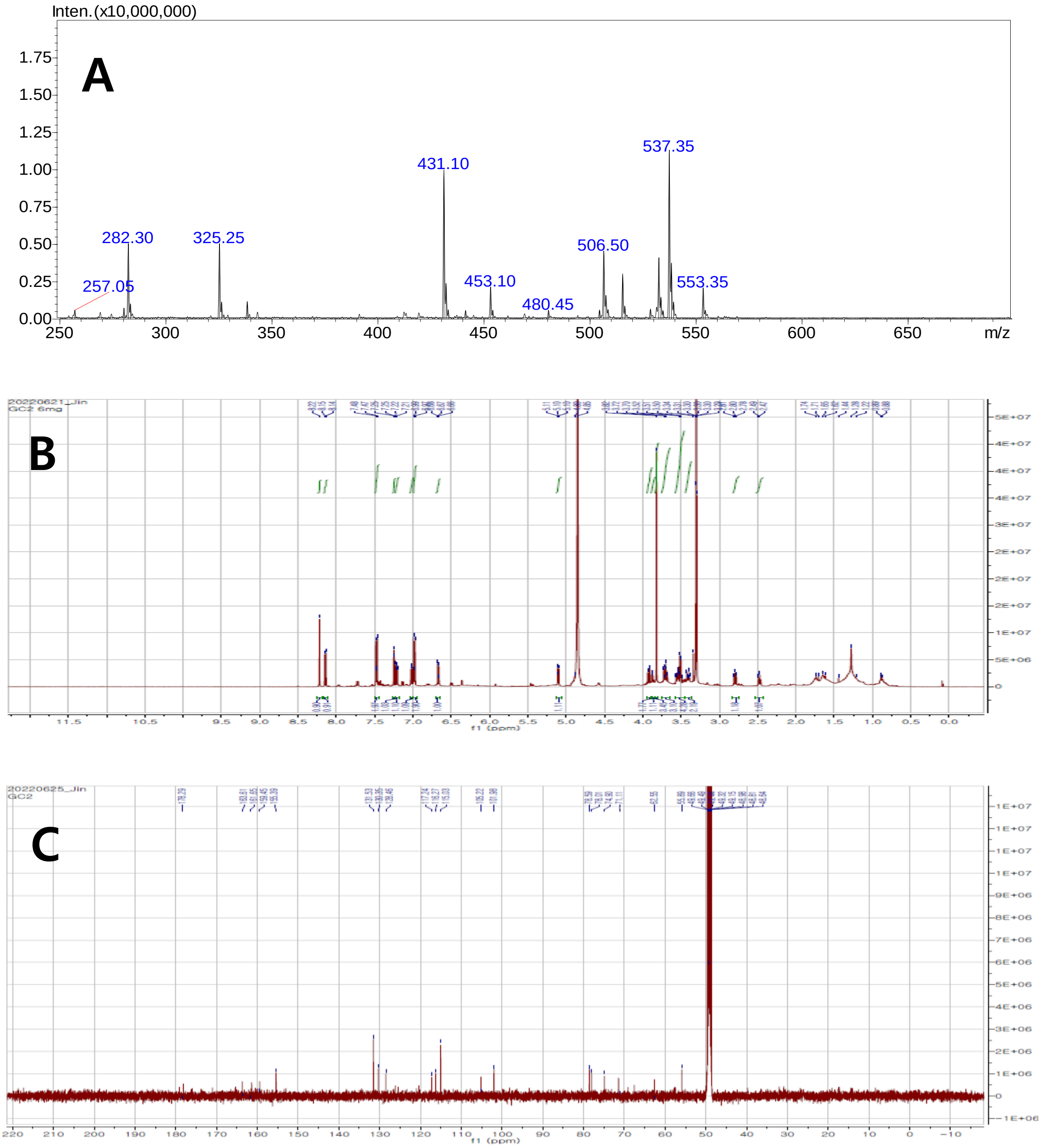

Figure S8. LC-MS(A), ^{1}H NMR(B) and ^{13}C NMR(C) spectra of compound **8** in MeOD-*d*$_4$ (400 MHz for ^{1}H NMR, 100 MHz for ^{13}C NMR)

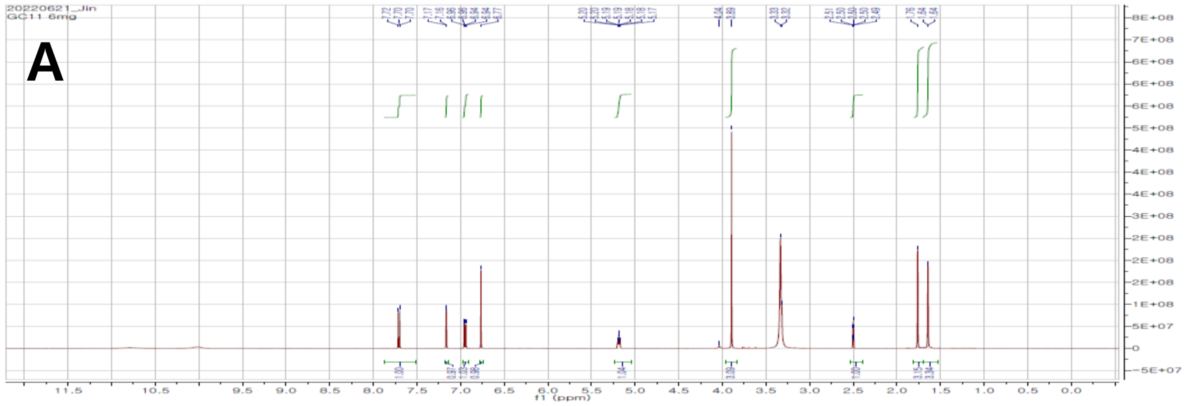

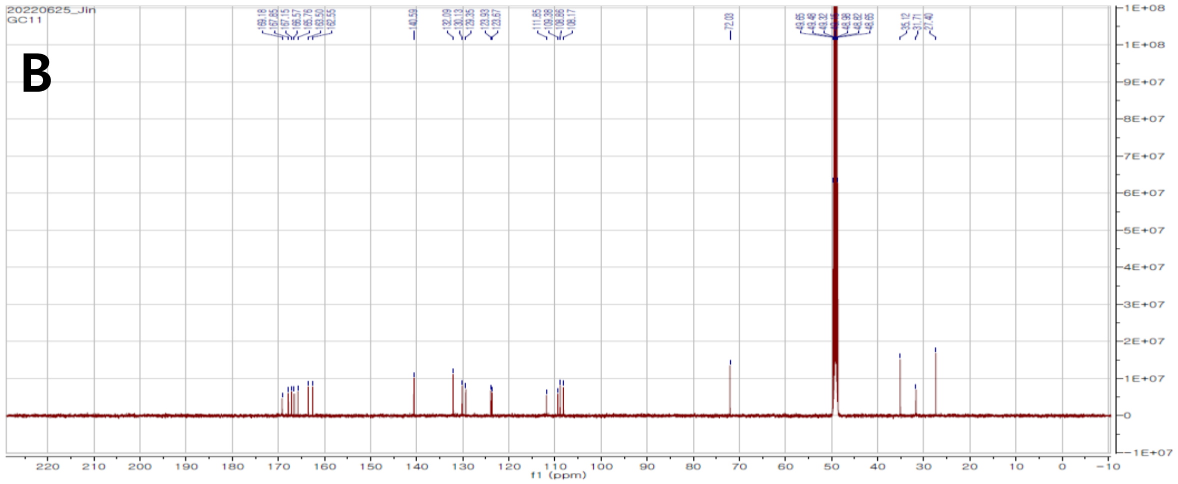

Figure S9. ^{1}H NMR(A) and ^{13}C NMR(B) spectra of compound **9** in MeOD-d_4 (400 MHz for ^{1}H NMR, 100 MHz for ^{13}C NMR)

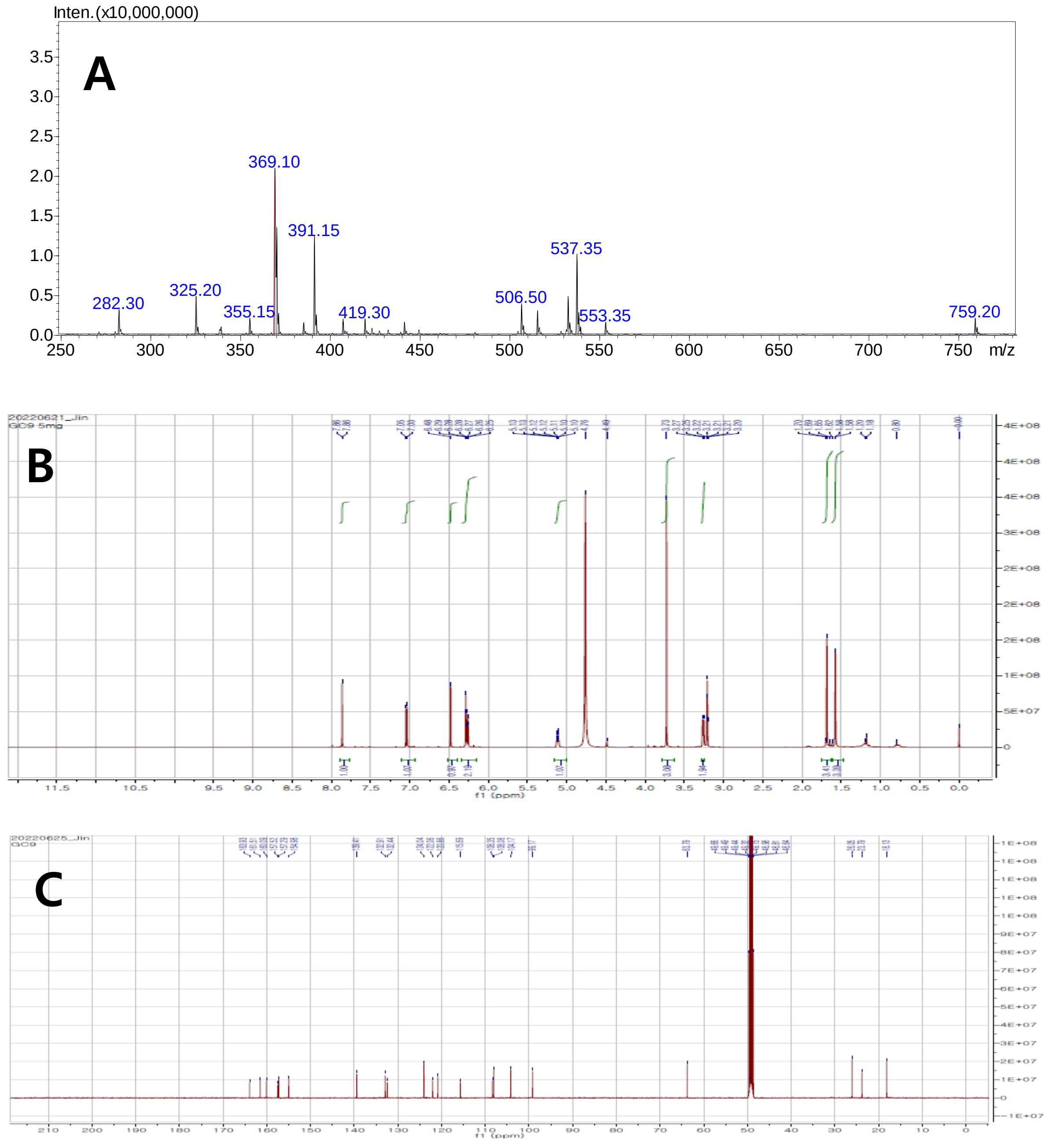

Figure S10. LC-MS(A), ^{1}H NMR(B) and ^{13}C NMR(C) spectra of compound **10** in MeOD-*d*$_4$ (400 MHz for ^{1}H NMR, 100 MHz for ^{13}C NMR)

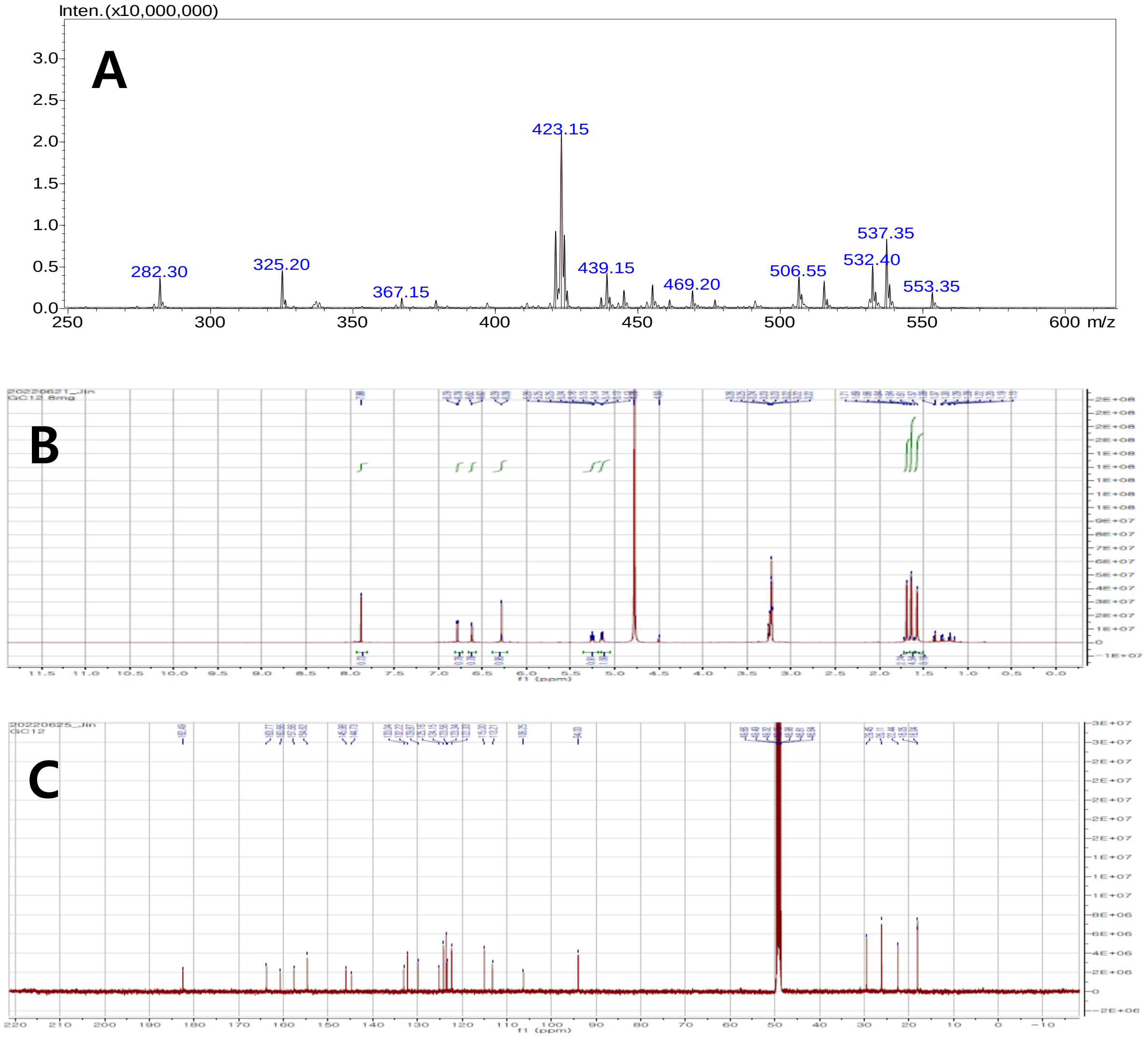

Figure S11. LC-MS(A), ^{1}H NMR(B) and ^{13}C NMR(C) spectra of compound **11** in MeOD-*d*4 (400 MHz for ^{1}H NMR, 100 MHz for ^{13}C NMR)
